# Do patients with Prader–Willi syndrome have favorable glucose metabolism?

**DOI:** 10.1186/s13023-022-02344-3

**Published:** 2022-05-07

**Authors:** Yanjie Qian, Fangling Xia, Yiming Zuo, Mianling Zhong, Lili Yang, Yonghui Jiang, Chaochun Zou

**Affiliations:** 1 Department of Endocrinology, The Children’s Hospital of Zhejiang University School of Medicine, National Clinical Research Center for Child Health, No 3333 Binsheng Road, Hangzhou, 310051 China; 2 Department of Genetics, Yale University School of Medicine, New Haven, USA

**Keywords:** Prader–Willi syndrome, Insulin, Insulin resistance, Adipose, Hormones

## Abstract

**Background:**

In recent years, more studies have observed that patients with Prader–Willi syndrome have lower insulin levels and lower insulin resistance than body mass index-matched controls, which may suggest protected glucose metabolism.

**Method:**

The PubMed and Web of Science online databases were searched to identify relevant studies published in the English language using the terms “Prader–Willi syndrome” with “glucose”, “insulin”, “diabetes mellitus”, “fat”, “adipo*”, “ghrelin”, “oxytocin”, “irisin” or “autonomic nervous system”.

**Results:**

The prevalence of impaired glucose intolerance, type 2 diabetes mellitus and some other obesity-associated complications in patients with Prader–Willi syndrome tends to be lower when compared to that in general obesity, which is consistent with the hypothetically protected glucose metabolism. Factors including adipose tissue, adiponectin, ghrelin, oxytocin, irisin, growth hormone and the autonomic nervous system possibly modulate insulin sensitivity in patients with Prader–Willi syndrome.

**Conclusion:**

Although lower insulin levels, lower IR and protected glucose metabolism are widely reported in PWS patients, the causes are still mysterious. Based on existing knowledge, we cannot determine which factor is of utmost importance and what are the underlying mechanisms, and further research is in urgent need.

## Introduction

Prader–Willi syndrome (PWS) is a rare and severe neurodevelopmental disorder resulting from the absence of expression of the paternal chromosome 15q11.2-q13.1 [1]. This syndrome is mainly described as marked obesity, severe hyperphagia, short stature, cryptorchidism, as well as mental retardation; PWS is the most common genetic and syndromic cause of obesity [1]. However, despite their severe obesity, PWS children and adults are generally reported to have significantly lower circulating insulin levels and lower insulin resistance (IR)/higher insulin sensitivity (IS) compared to those of body mass index (BMI)-matched obese controls [2]. That said, scant data exist for PWS infants. Moreover, several studies showed no differences in insulin or IR in obese PWS patients and controls [3, 4]. Low insulin levels and IR are thought to be protective factors against obesity-associated complications. Accordingly, one may hypothesize that PWS patients have favorable glucose metabolism compared to general obesity. However, is there sufficient evidence to prove this hypothesis? Furthermore, if PWS patients own favorable glucose metabolism, what may be the causes? This review describes existing knowledge about glucose metabolism in PWS and tries to identify possible factors modulating IR in PWS patients.

## IR and obesity-associated complications in PWS

Type 2 diabetes mellitus (T2DM) is a common obesity-associated complication in PWS (Table 1). As in the general population, obesity status plays a major role in T2DM and in metabolic syndrome development in PWS [3–7]. Without appropriate treatment, PWS patients can develop severe obesity and subsequently can develop T2DM at quite a young age. A mean age of 20 years of onset of T2DM in PWS patients was reported by Butler et al. [8]. Some studies even observed that PWS patients developed T2DM in the early teenage years [9]. However, the prevalence of impaired glucose intolerance, T2DM or metabolic syndrome in PWS tends to be lower when compared to that in obese controls (as presented in Table 1) [4, 10, 11]. Thus, there seem to be protective factors for glucose metabolism in PWS. The deduction that PWS patients have lower IR than BMI-matched controls is common in much of the published literature (Table 2). Some studies observed lower fasting or post-prandial insulin levels but intact glucose levels in PWS [12–14]. Others used HOMA IR index (HOMA-IR), HOMA IS index, QUICKI, or the log Matsuda index to prove lower IR in PWS [2, 15, 16]. One study calculated the cutoff values of HOMA-IR for PWS and made the conclusion that PWS patients were more sensitive to insulin than non-syndromic T2DM patients [17]. Because IR is an important risk factor for developing T2DM, the relatively lower IR may prevent T2DM in PWS patients.

**Table 1:** Comparison of prevalence of part of obesity-associated complications between PWS patients and controls

References	PWS patients				Obese controls			
	N	Mean age (years)	T2DM/IGT (%)	Other (%)	N	Mean age (years)	T2DM/IGT (%)	Other (%)
Greenswag [142]	232	23	T2DM (19%)					
Tauber et al. [143]	28		T2DM (7%)					
Butler et al. [144]	108	18.7	T2DM (14%)					
Krochik et al. [11]	75	8.4	T2DM (0%)		395	10.7	T2DM (1.5%)	
Thomson et al. [145]	30		T2DM (13.3%)					
Sinnema et al. [146]	102		T2DM (17%)					
Sinnema et al. [147]	12	57.8	T2DM (50%)					
Grugni et al. [4]	87	26 (P50)		MS (41.4%)	85	28 (P50)		MS (45.9%)
Bedogni et al. [18]	20	30		NAFLD (25%)	27	33		NAFLD (59%)
Fintini et al. [10]	21	12.4	IGT (14.3%)		42	12.5	IGT (21.4%)	
Fintini et al. [5]	274	20.3	T2DM (13.5%), IGT (10.2%)					
Yang et al. [17]	211		T2DM (13.7%)					
Damen et al. [148]	43		T2DM (5.1%)					

*T2DM* type 2 diabetes mellitus, *IGT* impaired glucose tolerance, *MS* metabolic syndrome, *NAFLD* non-alcoholic fatty liver disease

**Table 2:** Comparison of glucose metabolism between PWS patients and obese controls

References	matching factors	PWS patients			Controls	Glucose metabolism
		N	Mean age (years)	Mean BMI (kg/m^2^)	Mean BMI (kg/m^2^)	
Schuster et al. [149]	Age, BMI	9	11.5	35.5	35.1	During OGTT, lower fasting, peak, and AUC insulin in PWS; no differences in fasting, peak, and AUC glucose
Schuster et al. [149]	Age, BMI	14	33	42	39	During OGTT, no differences in fasting glucose or insulin and AUC glucose or insulin
Talebizadeh and Butler [26]	Age, BMI	23	22.7	36.5	38.1	Lower fasting insulin and higher IS in PWS; no differences in fasting glucose
Krochik et al. [11]	BMI	75	8.4	30.08	30.5	Lower fasting insulin, HOMA β-cell and higher IS, no differences in fasting glucose, 120-min glucose, and insulin index
Crino et al. [150]	Age, BMI	16	6.4	25.6	28	Lower fasting glucose, insulin and higher IS in PWS
Haqq et al. [13]	Age, BMI-Z	14	11.35			Lower fasting insulin and higher IS in PWS, no differences in the insulinogenic or disposition indices
Park et al. [81]	Age, BMI, PBF	15	11.2	24.8 (PBF 42.3)	26.3 (PBF 41.4)	Lower HOMA-IR in PWS, no differences in WBISI and fasting insulin
Brambilla et al. [3]	Age, BMI	50	11	32.5	29.6	Lower fasting glucose in PWS, no differences in fasting insulin and IS
Sohn et al. [151]	Age, BMI	30	7.05	19.9	21.8	Higher IS in PWS
Viardot et al. [23]	Age, PBF, Abdominal fat mass	12	27.9	39 (PBF 49)	34.3 (PBF 43.1)	No differences in fasting glucose, insulin, IS and HOMA-β
Faienza et al. [152]	Age, BMI	29	10.4	28.6	28.5	Lower fasting glucose, insulin and higher IS in PWS
Purtell et al. [22]	Age, BMI, PAF	10	27.9	37.0 (PAF 46.3)	34.3 (PAF 46.3)	No differences in IS and HOMA-β
Goldstone et al. [12]	Age, BMI	42	2.72	18.1	16.7	No differences in fasting insulin and IS
Bedogni et al. [18]	Age, PBF	20	30	39 (PBF 54)	42 (PBF 53)	No differences in fasting and 120-min glucose, IS and β-cell function
Fintini et al. [10]	Age, BMI	21	12.4	28.6	30.7	Lower fasting glucose, 120-min insulin, higher IS in PWS, no differences in fasting insulin and 120-min glucose
Hirsch et al. [16]	Age, BMI	22	28.7	29.2	25.7	Lower fasting glucose, insulin, and higher IS in PWS
Irizarry et al. [153]	Age, BMI-Z	14	10.9			Lower fasting insulin and higher IS in PWS, no differences in fasting glucose
Lacroix et al. [25]	Age, PBF, diabetic status	42	25.5	44.4 (PBF 52.2)	49.9 (PBF 50.5)	Lower fasting glucose, insulin, and higher IS in PWS
Purtell et al. [14]	Age, BMI, PAF	11	27.5	37.35 (PAF 46.53)	34.21 (PAF 46.25)	No differences in IS and HOMA-β
Mai et al. [115]	Age, BMI, PBF	30	35.7	45.5 (PBF 50.4)	46.8 (PBF 49.6)	Lower fasting insulin, C-Peptide, higher IS in PWS, no differences in fasting glucose
Paolo et al. [154]	Age, BMI	89	28.4	35.1	34.2	No differences in fasting insulin and IS

*BMI* body mass index, *BMI-Z* body mass index z-scores, *PBF* percent body fat, *PAF* percent abdominal fat, *OGTT* oral glucose tolerance test, *AUC* the areas under the curves, *IS* insulin sensitivity, *HOMA-β* homeostasis model assessment-insulin secretion, *HOMA-IR* homeostasis model assessment-insulin resistance, *WBISI* whole-body insulin sensitivity index

The prevalence of other obesity-associated complications is also lower in PWS patients. For example, the prevalence of non-alcoholic fatty liver disease (NAFLD) in women with PWS was significantly lower than in non-PWS women matched based on percent body fat (PBF) [18]. Fintini et al. [10] reported that G2 stage of NAFLD was significantly less frequent in PWS children than in BMI-matched peers. The prevalence of coronary artery disease also appears to be lower in PWS than in simple obesity [19]. Because IR also plays an important role in the development of NAFLD and coronary artery disease, it is very likely that the lower IR leads to the lower prevalence of NAFLD or coronary artery disease in PWS [20, 21]. However, whether these conclusions are applicable to the majority of PWS patients needs more study.

## Factors modulating IR in PWS

### Adipose tissue and IR in PWS

A close association between adipose tissue (AT) and IR in PWS patients was reported. Lower circulating insulin levels and lower IR in PWS compared to obese controls were described in most studies, but a few exceptions existed. Purtell et al. [14, 22] found no differences in HOMA-IR, HOMA-β, and insulin secretion rate in comparisons between PWS patients and abdominal fat mass-matched obese controls. Other studies reported equally increased HOMA-IR and HOMA-β in PWS and obese controls who were matched in BMI, PBF, and total body and central abdominal fat mass [23]. These differences may be associated with the fact that in these studies the PWS and the control groups were matched based on parameters of body AT including accurate parameters of fat ratio distribution [18]. Owing to the special characteristics of body AT in PWS, the body fat patterning of PWS may be completely different from that of simply BMI-matched controls. If alterations in AT cause the lower IR in PWS, it is not surprising that these studies failed to find lower IR in PWS.

Fat distribution is quite peculiar in PWS and can be summarized using three major characteristics. First, increased fat mass and decreased lean mass have been widely reported in PWS children and adults compared to BMI-matched controls [1]. In the young underweight PWS children, both skinfold (subscapular and tricep -) standard deviation scores for BMI and BMI-adjusted leptin levels were elevated, suggesting excess adiposity may begin early in PWS infants, long before the onset of obesity [24, 25]. It was reported that the increased fat/lean mass ratio persisted even if normal weight was achieved in PWS patients [26]. Second, PWS patients have relatively lower visceral adipose tissue (VAT) and higher subcutaneous adipose tissue (SAT) compared to BMI-matched controls, though with some disputes [1]. In one subtype of obesity named “metabolically healthy but obese individuals” VAT is also significantly lower compared to another subtype named “metabolically abnormal obese individuals” [27]. Third, appendicular fat mass is increased while trunk fat mass is decreased in PWS adults [1, 28]. However, data about VAT or SAT and appendicular or trunk fat mass are lacking in PWS infants, making it difficult to determine if PWS patients are born with these characteristics.

#### Characteristics of adipose tissue (AT) and IR in PWS

The relationship between AT and glucose metabolism in PWS remains largely unknown. According to Talebizadeh et al. [26], PWS subjects had larger volume and fewer numbers of adipocytes than non-PWS obese controls. Others found the measured adipocyte size was higher than the theoretical adipocyte size in PWS, suggesting a tendency for PWS to develop larger adipocytes [25]. However, in the general population, the presence of large adipocytes seems to be an indicator of a poor adipogenic ability of AT [29], and it may serve as a risk marker for developing T2DM [30]. Larger adipocytes have increased fat storage and decreased concentration of transporter distribution in each fraction, which may decrease their efficiency for behaving as “metabolic buffers” and thus may lead to IR [30]. Another possible explanation is that larger adipocytes cause a failure to recruit new adipocytes, thus diminishing the expandability of AT and resulting in IR by a lipotoxic mechanism [31].

**Fig. 1:**
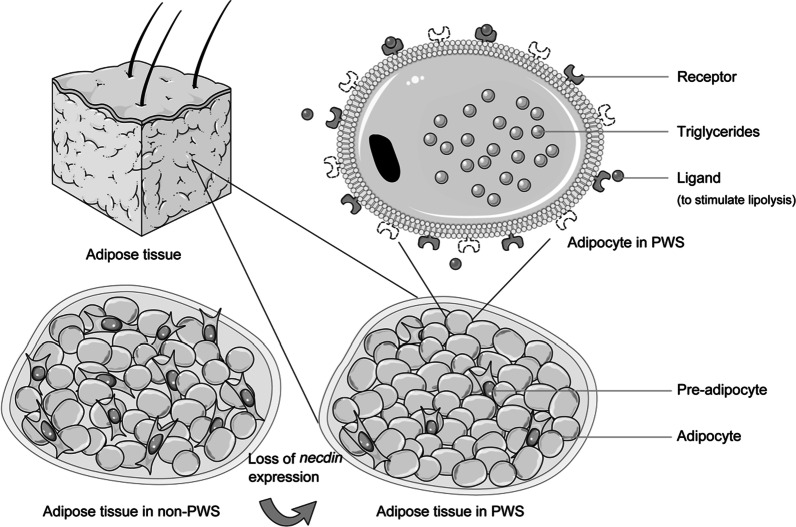
The hypothesized mechanism underlying the effects of loss of *necdin* expression on adipose tissue in PWS. Adipogenic differentiation is promoted, thus leading to lower pre-adipocyte and higher adipocyte content. Adipocytes of PWS patients are insensitive to lipolytic stimulation, leading to accumulation of triglycerides

When talking about adipocyte proliferation and differentiation, *necdin*, one important gene located in PWS region, must be discussed. By studying pre-adipocytes, researchers found that over-expressing *necdin* inhibited adipogenesis, whereas downregulating *necdin* promoted adipogenic differentiation [32, 33]. Treated with adipogenic inducers, adipose stromal-vascular cells derived from *necdin*-null mice differentiated into more adipocytes than those from wild-type mice [34]. This was verified to some extent in vivo because *necdin*-null mice had more fat mass compared to controls, which was attributed to adipocyte hyperplasia [34]. In addition, pre-adipocyte content was lower in the stromal vascular fraction of AT in PWS, and adipocytes of PWS patients were insensitive to lipolytic stimulation [33]. Lower pre-adipocyte content may owe to an activated adipogenic process, and impaired lipolytic response may lead to triglycerides accumulation [33]. Thus, loss of *necdin* expression in the AT of PWS patients may explain the increased fat mass in PWS [25, 33] (Fig. 1). As downregulating *necdin* promotes adipogenic differentiation, one assumption is that those “relative larger adipocytes” (which need further verification) are actually well differentiated and are markers of strong expandability of AT in PWS instead. According to the AT expandability hypothesis, ectopic lipid deposition occurs when individuals reach their AT expansion limits, which are determined by genetic and environmental factors [31]. However, ectopic lipid deposition (e.g., fat accumulation in VAT or liver) is believed to aggravate IR [35]. Owing to the probably stronger expandability of AT, PWS subjects have relatively larger adipocytes and increased fat accumulation, which do not synchronize with IR despite severe obesity. Researchers once reported “metabolically healthy but obese individuals” had two- to three-fold higher expressions of genes associated with adipocyte differentiation, though the proportion of small adipocytes was actually lower than “metabolically abnormal obese individuals”, which may share some similarities with PWS patients [36].

Others suggest a better metabolic environment of AT in PWS. Many genes associated with IR are downregulated in the AT of PWS subjects [25]. In AT of PWS, candidate genes encoding proinflammatory markers are also underexpressed, which is beneficial to glucose metabolism [25]. These results seem to be consistent with the hypothetically better glucose metabolism in PWS.

#### Fat distribution and IR in PWS

In 2001 Goldstone et al. [37] first reported that females with PWS had significantly lower VAT than obese controls, whereas there were no differences in BMI, total AT, or PBF. The authors also found that lower fasting insulin levels, lower insulin/glucose ratio, and higher C-peptide/insulin ratio were associated with VAT rather than total or abdominal SAT [37]. Today, quite a few studies support significantly lower VAT in PWS [1], but the correlation between lower VAT and lower IR in PWS is still vague.

Fat accumulation in VAT is an important risk factor for developing IR and obesity-associated complications in the general population [35, 38]. VAT may have more fatty acid accumulation and more actively lipolytic activities than SAT [39]. Increased VAT could expose the liver to excessive fatty acids and glycerol via portal lipid flux, and then cause increased hepatic glucose and triglyceride production and decreased insulin clearance, thus leading to hepatic IR [35, 39, 40]. The microenvironment of VAT also differs from that of SAT [41]. Implantation of adipocytes into VAT in nude mice caused increased IR, while surgical removal of VAT improved IS, but this was not the case for SAT [42]. More proinflammatory cytokines, such as tumor necrosis factor-α (TNF-α) and interleukin-6, were released by macrophages in VAT, thus increasing IR [42]. Twenty genes related to fat and glucose metabolism, including PPAR-γ and adiponectin gene, were markedly different in VAT and SAT [42]. On the other hand, researchers have observed an important relationship between SAT and IR in the general population [43–46]. Functioning as a buffer for daily lipid fluxes, larger SAT may help to prevent from fatty acid-induced IR [33]. Impaired SAT may indirectly deteriorate IR via VAT or hepatic AT [29, 42]. Lower VAT and higher SAT may play a role in regulating IR in PWS.

Decreased truncal and increased appendicular fat mass result in a decreased truncal/peripheral fat ratio in PWS adults. In general obesity, pioglitazone improved IR and caused a lowered waist-to-hip ratio via increasing lower body AT without changing VAT in general obesity [47]. It was once reported patients with T2DM had increased truncal/peripheral skin folds thickness ratios compared to controls, while their intraperitoneal fat mass was at the same level [43]. Larger thigh subcutaneous fat mass may correlate with better glucose and fat metabolism, and the decreased truncal/peripheral fat ratio may contribute to lower IR in PWS [48, 49].

### Hormones, peptides and IR in PWS

#### Adiponectin and IR in PWS

Some studies reported that circulating adiponectin levels in PWS patients were significantly higher than those in BMI-matched controls and lower than those in lean controls [13, 50–52]. Haqq et al. [13] also detected levels of isoforms of adiponectin and found high molecular weight (HMW) adiponectin levels and found that the HMW/total adiponectin ratio was increased compared to that of BMI-matched controls. Compared to controls, total and HMW adiponectin levels were elevated in female *Magel2*-null mice, who maintained IS despite their increased adiposity [53]. Several studies have observed that adiponectin levels are negatively correlated with IR in PWS [13, 52]. It is likely that higher adiponectin levels cause lower insulin levels and lower IR in PWS.

Adiponectin may be a potential insulin enhancer. The ability of insulin of sub-physiological levels to suppress glucose production was improved by adiponectin administration in isolated hepatocytes [54]. Adiponectin can alleviate IR in mouse models that developed it for high-fat feeding, leptin-receptor deficiency or agouti overexpression [55]. IR also was partially ameliorated in globular domain adiponectin transgenic (gAd Tg) ob/ob mice compared with ob/ob mice, though AT levels were actually increased in gAd Tg ob/ob mice [56, 57]. HMW adiponectin or the ratio of HMW to total adiponectin may perform a major role [13, 58]. In the general population, a decrease in circulating adiponectin levels at baseline preceded a decrease in IS in a prospective study [59]. A Mendelian randomization study reported that both genetically determined and actually observed adiponectin increased IS in Swedish men, a result partially explained by BMI and waist circumference [60].

In PWS, whether and how adiponectin affects IR remain largely unknown. Several studies have found correlations between adiponectin and fat distribution in PWS patients. Adiponectin was negatively correlated with VAT [19, 38], or tended to be inversely correlated with PBF or BMI in PWS [61]. Kennedy et al. [52] stated that adiponectin had an inverse relation to waist-to-hip ratio in PWS. In the general population, the expression level of adiponectin was higher in SAT than in VAT [39, 46, 62]. Increased adiposity of gAd Tg ob/ob mice mainly was attributed to fat accumulation in SAT rather than in VAT or liver when compared to ob/ob mice [56, 63]. After administration of gAd, expressions of molecules involved in fatty-acid influx into the liver was downregulated in lipoatrophic mice [55]. Perhaps adiponectin affects glucose metabolism by altering fat distribution in PWS.

**Fig. 2:**
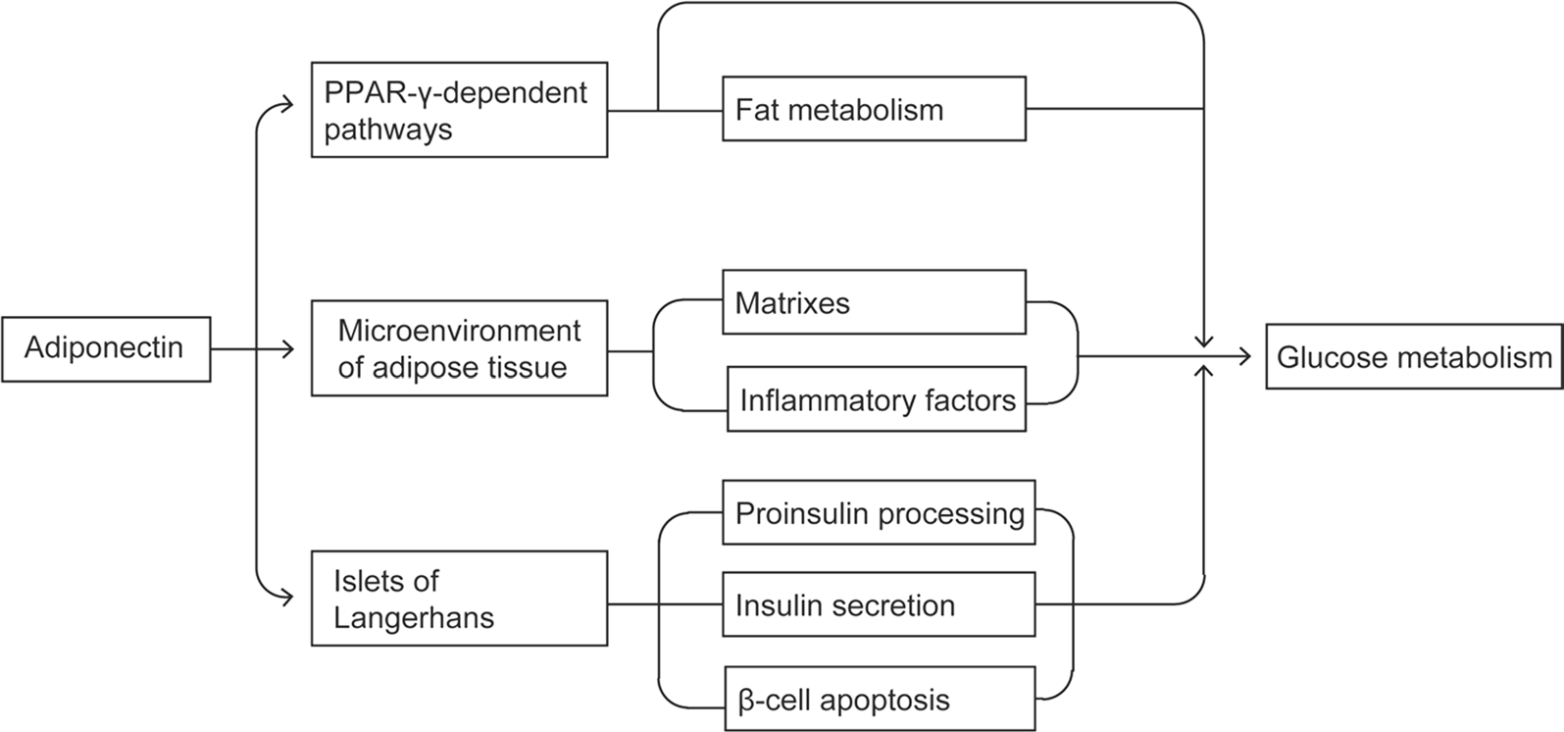
The hypothesized model of the effects of adiponectin on glucose metabolism. Adiponectin may affect PPAR-γ-dependent pathways, microenvironment of adipose tissue and islets of Langerhans, thus modulating glucose metabolism. But the relations and mechanisms remain largely unknown

Adiponectin itself may exert important effects on glucose metabolism directly or indirectly (Fig. 2). Adiponectin can alter the process of tyrosine phosphorylation of insulin receptors in skeletal muscle [59]. By binding to its receptors, adiponectin regulates molecular pathways involving AMPK, PPAR-α, PPAR-γ and others [56, 63, 64]. In turn, mRNA levels in WAT and circulating levels of adiponectin as well as IS were significantly increased by rosiglitazone (a PPAR-γ agonist), though more fat was accumulated [55]. In lipoatrophic mice whose PPAR-γ/RXR activity was severely reduced, serum adiponectin was undetectable and IR was developed, which can be ameliorated by administration of adiponectin [55]. Because PPAR-γ is crucial for glucose metabolism and for differentiation of adipocytes [65], relatively high adiponectin levels may be associated with better glucose metabolism and with adipogenic differentiation in PWS.

Adiponectin may modulate IR by affecting the microenvironment of AT or other organs. Adiponectin has the potential to work as a matrix-forming protein because it has striking structural homology to collagens VII and X [66]. The production and action of TNF-α can be reduced by adiponectin [56, 66, 67]. High adiponectin levels in PWS may contribute to a good glucose and fat metabolic microenvironment, whereas low adiponectin levels in general obesity may be a reflection of microenvironmental dysfunction and an “unhealthy” AT expansion [63].

In addition, some studies have shown a relationship between adiponectin and pancreatic function. A negative correlation was found between adiponectin and the proinsulin/insulin ratio, which was a marker of β-cell failure in the general population [68]. Children with type 1 diabetes mellitus were reported to have significantly higher circulating adiponectin levels than controls [69]. Receptors of adiponectin are markedly expressed in β-cells, and adiponectin has the ability to promote glucose-stimulated insulin secretion and to prevent apoptosis of β-cells in vitro [63]. If the pancreatic function is impaired in PWS, adiponectin levels may be elevated by compensatory mechanisms.

#### Ghrelin and IR in PWS

As first reported by Cummings et al. in 2002 [70], the orexigenic hormone ghrelin has received wide attention because it elevates remarkably in PWS patients compared to obese controls or even lean controls. Some studies state hyperghrelinaemia can be observed at all ages of PWS, including infants [71, 72]. Elevated levels of ghrelin may occur before the onset of hyperphagia and obesity [71, 73, 74], although some disagreements exist [73, 75]. Cleaved from proghrelin, acyl ghrelin (AG) and desacyl ghrelin (DAG) coexist in the human circulatory system. Interestingly, some researchers noticed PWS children had a significantly higher AG/DAG ratio than healthy controls, which was comparable to obese controls [76]. But the high AG/DAG ratio was attributed mainly to high AG levels in PWS and to low DAG levels in obese controls [76]. However, other research studying PWS infants and *Magel2*-null mice found that they had normal AG but high DAG levels, leading to a lower AG/DAG ratio than controls [72, 77].

Surprisingly, researchers found a negative correlation between ghrelin and insulin levels and HOMA-IR in PWS [71, 78, 79], even in PWS infants [77]. Others found fasting AG and DAG levels were positively related to whole-body IS index [80]. The areas under the curves of AG were negatively related to the areas under the curves of insulin in PWS [81]. In PWS patients, groups without glucose intolerance had a significantly higher AG/DAG ratio than those with glucose intolerance [79]. Thus, one possibility is that altered ghrelin levels account for lower insulin levels and IR in PWS.

In the general population, a negative relationship also was observed between ghrelin and insulin levels and IR [82, 83]. However, many studies stated that ghrelin inhibited insulin secretion both in humans and in animals and was accompanied by increased glucose levels and impaired glucose tolerance [15, 84–86]. Besides, higher IS was observed in mice deficient in ghrelin or its receptors [85, 87, 88]. Other research reported ghrelin was able to stimulate insulin secretion [89]. It is possible that ghrelin affects IR differently in different conditions. Alternatively, AG or DAG levels, as opposed to total ghrelin levels, may play an important role in glucose metabolism. The effects of AG and DAG on glucose metabolism seem to be at opposite poles. Research found that AG reduced insulin secretion in humans or isolated islets, and a large dose of AG caused IR [82, 90–92]. In growth hormone secretagogue receptor-knockout mice, DAG regulated expression of genes involved in glucose and lipid metabolism in AT, muscle, and liver [93]. DAG administration also was reported to markedly improve IR in rodents, healthy volunteers and patients with T2DM [91, 94–96]. Many studies proposed that the action of DAG depended at least in part on antagonizing the action of AG [82, 89, 90, 97]. However, the effects of AG and DAG on glucose metabolism in PWS are still unclear. Some experiments suggested ghrelin can regulate the pancreas directly. Both AG and DAG stimulated proliferation and prevented apoptosis of HIT-T15 β-cells [98]. DAG can even rescue β-cells from streptozotocin-induced β-cell damage [85].

Some researchers found a negative correlation between ghrelin and BMI, BMI percentile, and VAT in PWS, suggesting ghrelin may regulate AT in PWS [78, 99, 100]. When studying the relationship between ghrelin and IR in PWS, the confounding effects of fat patterning should be taken into consideration.

#### Oxytocin levels and IR in PWS

Several studies have investigated alterations of oxytocin (OXT) in PWS patients. Swaab et al. [101] found that immunoreactivity of OXT and the number as well as the volume of OXT-expressing neurons were significantly decreased in PWS patients compared to healthy controls. A reduction of OXT-producing neurons was observed in *Necdin*-deficient mice [102]. In the hypothalamus of *Magel2*-null mice, the immunoreactivity of OXT seemed stronger than controls, but the enhanced signal was attributed mainly to an accumulation of OXT intermediate forms, whereas expressions of OXT mature forms were actually decreased [103]. In *Magel2*^+m/−p^ mice, researchers observed that central OXT was decreased at birth and was increased in adulthood, which may owe to a compensatory mechanism [104]. Besides, OXT levels in the cerebrospinal fluid and the plasma were reported to be elevated in PWS patients compared to healthy controls [105, 106]. The expression of OXT receptor gene was deficient in lymphoblasts of PWS males [107].

The association between OXT and IR is seldom studied in PWS. In the general population and in rodents, OXT is closely related to glucose metabolism. OXT levels were reduced in patients with type 1 and type 2 diabetes mellitus [108]. And lower OXT levels were correlated to higher insulin levels, HOMA-IR and HbA1c levels in T2DM patients [108]. However, men with metabolic syndrome had higher OXT levels than those without metabolic syndrome [108]. OXT administration can either improve or aggravate IR in humans, probably depending on the dose or the route of administration [108–110]. Oxytocin-deficient and high fat diet fed OXT receptor-deficient (Oxtr^−/−^) mice to develop IR and glucose intolerance [109]. OXT administered via intravenous injection or via injection into the brain’s third ventricle improved IR in diabetic or prediabetic mice [109, 111]. There were also reports claiming a deteriorating effect of OXT on glucose metabolism in rodents [108, 109, 111].

OXT seems able to modulate the pancreas directly. OXT was detected in human and rat pancreatic extracts, and the levels were higher than plasma OXT levels [112]. In addition, central nervous system OXT receptors also are distributed in the pancreas, adipocytes, anterior pituitary gland, vagus nerve and gastrointestinal tract [108]. OXT was reported to stimulate insulin secretion in islets or in β-cells, both in vivo and in vitro [110]. Furthermore, OXT has protective effects on islets by promoting proliferation and by inhibiting apoptosis of β-cells [113]. The pancreas of streptozotocin-induced diabetic rats can be improved histologically and functionally by OXT administration [114]. It is possible that OXT is central to the functional or even to histological changes in pancreas in PWS patients.

#### Irisin and IR in PWS

Irisin is a myokine mainly derived from muscle and functions in inducing the browning of white AT [115]. In recent years, studies have reported that irisin is correlated with insulin and HOMA-IR in rodents and human [116–118]. Irisin intervenes in the process of apoptosis in the pancreatic islets [16, 119]. While Hirsch et al. [16] observed significantly higher levels of salivary irisin in PWS patients than in normal-weight controls, Mai et al. [115] subsequently found circulating irisin levels were significantly lower in PWS patients than in BMI-matched controls. They also observed a positive correlation between irisin and %FM, insulin and HOMA-IR in PWS [115]. This result is quite interesting because irisin is proved also to be an adipokine and is mainly secreted by SAT [120]. However, because PWS patients have lower muscle mass and less exercise than obese controls, both of which contribute to lower irisin, it is presently difficult to conclude whether changes of fat patterning play a role in regulating irisin levels in PWS [115].

#### Growth hormone and IR in PWS

Growth hormone (GH) is capable of stimulating insulin secretion and is commonly deficient in PWS patients; therefore, one may hypothesize that GH deficiency (GHD) causes the lower insulin levels in PWS [121, 122]. However, low insulin levels and high IS are not generally observed in non-PWS children with GHD, and non-PWS adults with GHD even develop IR [123]. In non-PWS GHD adults, GHD is usually secondary to other primary diseases [124]. However, PWS patients may be born with GHD. In most PWS children, impaired intrauterine and postnatal growth rates are observed and GHD is diagnosed [1]. The possible congenital GHD in PWS is reminiscent of patients with isolated GHD (IGHD). Interestingly, IGHD patients also had low insulin levels and relatively low IR, though their β-cell function was reduced and their frequency of impaired glucose tolerance was increased [124, 125]. Patients with GH receptor deficiency also showed lower fasting insulin levels and lower IR than BMI-matched controls despite higher PBF [126]. In addition, Lit/lit mice (whose gene encoding the GH releasing hormone-receptor is mutated), GH knockout (GHKO) mice, and GH receptor knockout (GHRKO) mice all had decreased insulin levels, increased IS and impaired glucose tolerance [121, 127]. Furthermore, both GHKO mice and GHRKO mice preferentially accumulated AT in SAT regions, and GHRKO mice had increased adiponectin levels, which shared some similarities with PWS patients [127, 128].

**Fig. 3:**
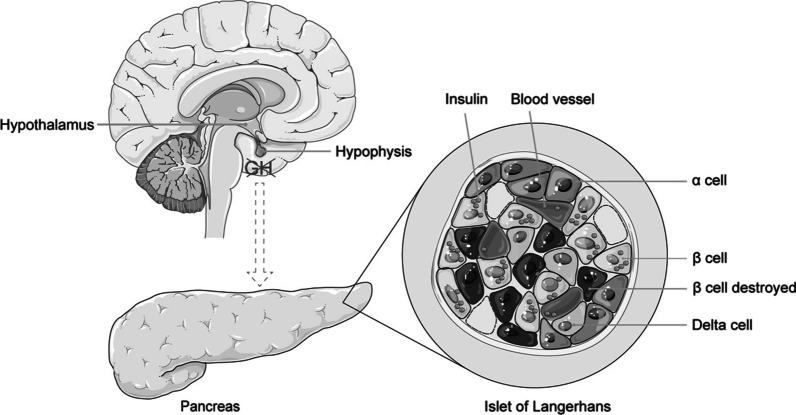
The hypothesized mechanism underlying the effects of GH on pancreatic islets. Without GH stimulation, the development and functional maintenance of islets are impaired. β-cell mass is reduced and destruction of β-cell is promoted. The insulin secretion function of β-cell is also impaired

Noticeably in both GHKO mice and GHRKO mice, islet size was significantly reduced [121, 127]. Markedly decreased β-cell mass also was observed in GHRKO mice [121]. It was interesting to find defective β-cell secretory function, decreased β-cell proliferation and reduced β-cell mass in high fat diet fed βGHRKO mice (whose GH receptors in β-cells were disrupted) [129]. Thus, it is possible that GH is critical for the development and functional maintenance of islets and that nonfunctioning GH signaling leads to impaired glucose metabolism. A primary defect in GH signaling may cause a primary defect in islets in PWS, thus leading to lower insulin levels (Fig. 3).

Although GHD itself has not been studied intensively in PWS, GH treatment is widely applied to PWS patients because it significantly improves obesity and comorbidities [1]. However, diabetogenic effects of GH are still observed in PWS patients treated with GH [2, 130]. One possibility is that late initiation of GH treatment provides no help for the irreversible defect in islets and therefore fails to improve glucose metabolism in PWS.

### Autonomic nervous system and IR in PWS

The autonomic innervation of islets and the effects of autonomic activation on hormone secretion in islets both in humans and rodents suggest the possible alteration of the autonomic nervous system (ANS) may account for the lower insulin levels and IR in PWS [131]. Activated peripheral nervous system promotes glucose-stimulated insulin secretion and thus attenuated peripheral nervous system can lead to lowered insulin levels [131]. A disturbance in the ANS is hypothesized in PWS patients [132, 133]. Some features of PWS, such as abnormalities in thermoregulation and sleep control and altered perception of pain indicates altered ANS. Decades ago, researchers found more patients with PWS had pupillary constriction of 2 mm or more, an abnormal 30:15 R-R interval ratio and changed diastolic blood pressure after standing compared to healthy controls [134]. Choe et al. [135] found a reduced gastric emptying in PWS, though their ghrelin levels were remarkably higher, which may be related to altered ANS.

ANS also may play a role in AT. Vagotomy upregulated the catabolic enzyme hormone sensitive lipase and downregulated insulin-dependent glucose uptake as well as FFA take, thus aggravating IR [136]. Different ANS innervation in VAT and SAT indicates alterations of ANS may affect fat distribution [132]. A correlation between signs of a high ratio of sympathetic vs. parasympathetic reactivity and VAT was found in the general population [137]. In addition, ANS is associated with both ghrelin and oxytocin. Vagotomy elevated plasma levels of ghrelin and inhibited the effects of ghrelin on reducing insulin secretion in rodents [78, 138]. Oxtr^−/−^ male mice had lower adrenalin levels than controls, but whether this altered ANS activity is associated with features of Oxtr^−/−^ male mice needs further research [139].

## Discussions and conclusions

**Fig. 4:**
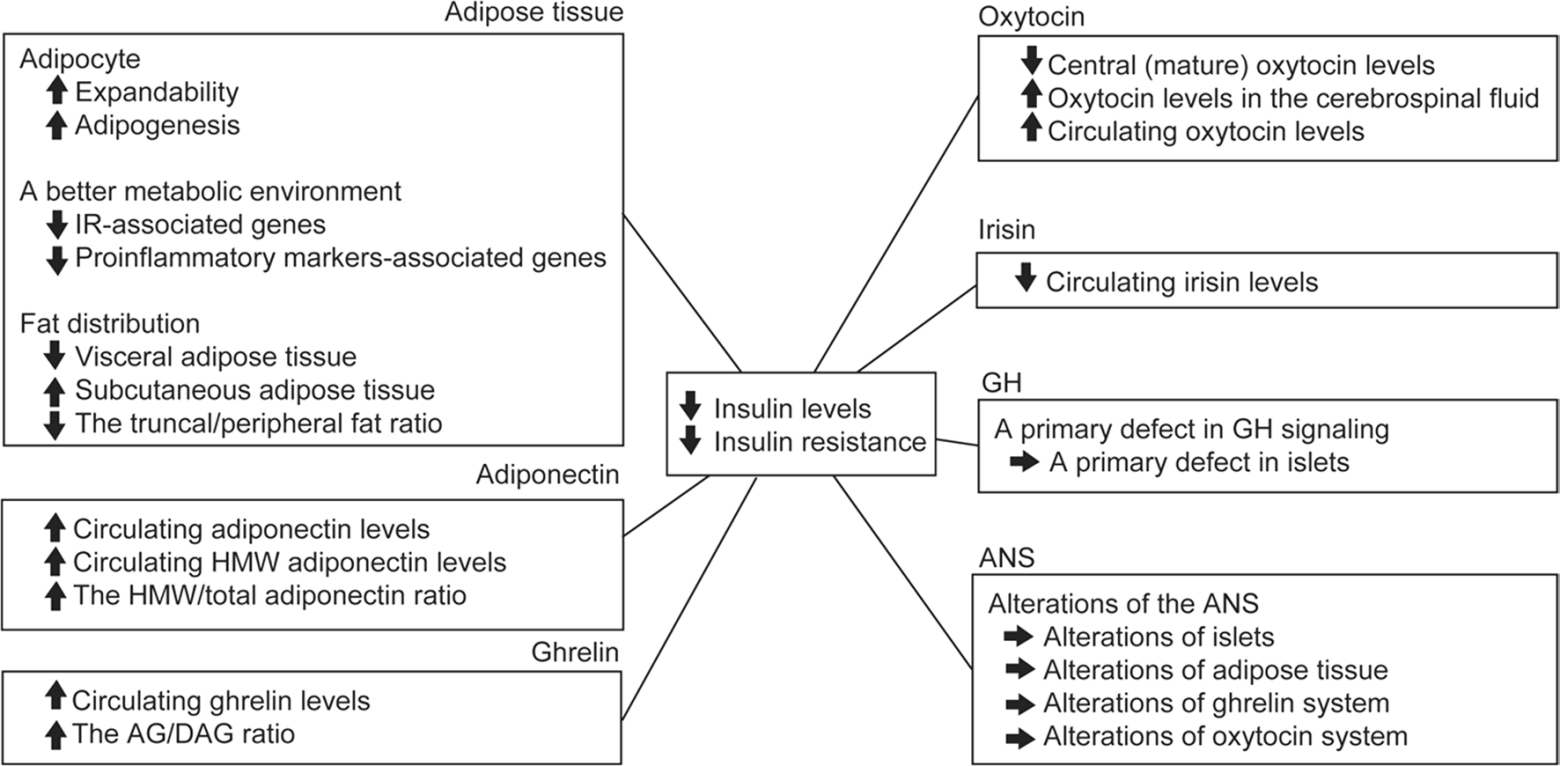
The summary figure of possible factors contributing to lower insulin levels and lower IR in PWS patients. “↑” means “increased”; “↓” means “decreased”; “→” means “leads or lead”. Adipose tissue, adiponectin, ghrelin, oxytocin, irisin, growth hormone and the autonomic nervous system all may play a role in the lower insulin levels and lower IR in PWS patients. *IR* insulin resistance, *HMW* high molecular weight, *AG* acyl ghrelin, *DAG* desacyl ghrelin, *GH* growth hormone, *ANS* autonomic nervous system

Many important questions remain. The jury is still out on whether the prevalence of T2DM or other obesity-associated complications in PWS is lower than in general obesity. Further population studies are needed. It seems that relatively lower IR protects glucose metabolism in PWS, and adipose tissue, adiponectin, ghrelin, oxytocin, irisin, growth hormone and ANS all may play a role in lower insulin levels and in lower IR in PWS patients (summarized in Fig. 4). But the causes and underlying mechanisms remain largely unknown. For example, do alterations of AT truly protect glucose metabolism in PWS? Researchers reported PWS patients had larger adipocytes, but the evidence is insufficient. The true relationships between large adipocytes and adipogenic potential and metabolic conditions also need further research. Adiponectin can affect IR in PWS patients, but does it behave as a causative role? Or does adiponectin regulate IR by altering fat distribution in PWS? Numerous studies exist on the correlation between ghrelin and insulin secretion, but the results are controversial. It is unknown whether altered ghrelin levels are correlated with impaired pancreatic function. OXT plays an essential role in glucose metabolism, but the lack of research about OXT and IR in PWS prevents us from further analyzing. Irisin may also play a role in lower insulin levels and in lower IR in PWS patients. Besides, does GHD lead to impaired development and functional maintenance of islets in PWS? ANS dysfunction may be central to the pathogenesis of PWS, but existing studies on this subject are very limited. Figuring out the underlying mechanism requires further research.

Despite probably favorable glucose metabolism compared to general obesity, PWS patients are still vulnerable to T2DM due to severe obesity. Thus, the regular monitoring of glucose homeostasis parameters is advised. Fasting glucose levels, hemoglobin A1c, lipid profile and evidence of microvascular complications and cardiovascular diseases should be investigated annually to predict the occurrence of T2DM in PWS. If PWS patients are diagnosed with T2DM, management should follow general guidelines as no systematic studies of diabetes management in PWS are available [1, 2]. The prevention of obesity should be the most important goal and lifestyle interventions including diets and exercise should be the first-line therapy. Dietary intake was associated with the gut microbiota in PWS; recently, our lab has found that the gut microbiota may play a role in lower insulin levels and in lower IR in Chinese PWS patients (unpublished data), consistent with the findings of Olsson et al. [140, 141]. Thus, intensive diet counseling may help improve IR and T2DM in PWS. The evidence of pharmacological treatment for T2DM in PWS is lacking. Case reports have suggested that anti-diabetic drugs including metformin, acarbose and exenatide are effective and safe in PWS [1, 2]. Thiazolidinediones, sulfonylureas and insulin are not always recommended owing to the treatment-related weight gain [2]. More data on the efficacy and safety of the existing or potent anti-diabetic drugs for T2DM in PWS are in urgent need since lifestyle interventions are difficult to achieve in all PWS patients especially during adolescence.

In conclusion, although lower insulin levels, lower IR and favorable glucose metabolism are widely reported in PWS patients, the causes are still mysterious. Altered adipose tissue, elevated adiponectin levels, changed ghrelin and oxytocin and irisin levels, GHD and impaired ANS all may play a role in lower insulin levels and in lower IR in PWS patients. Based on existing knowledge, we cannot determine which factor is of utmost importance and what are the underlying mechanisms. Further research is required because it can help us to better understand and then to improve glucose metabolism in PWS. What’s more, the research findings also may provide ideas and methods for general obesity to improve IR.

## Data Availability

Not applicable.

## Abbreviations

PWS: Prader–Willi syndrome
IR: Insulin resistance
IS: Insulin sensitivity
BMI: Body mass index
T2DM: Type 2 diabetes mellitus
HOMA-IR: HOMA IR index
NAFLD: Non-alcoholic fatty liver disease
PBF: Percent body fat
AT: Adipose tissue
VAT: Visceral adipose tissue
SAT: Subcutaneous adipose tissue
TNF-α: Tumor necrosis factor-α
HMW: High molecular weight
gAd Tg: Globular domain adiponectin transgenic
AG: Acyl ghrelin
DAG: Desacyl ghrelin
OXT: Oxytocin
Oxtr^−^^/^^−^: OXT receptor-deficient
GH: Growth hormone
GHD: Growth hormone deficiency
IGHD: Isolated growth hormone deficiency
GHKO: Growth hormone knockout
GHRKO: Growth hormone receptor knockout
ANS: Autonomic nervous system
